# Direct observations of pure electron outflow in magnetic reconnection

**DOI:** 10.1038/s41598-022-14582-3

**Published:** 2022-06-30

**Authors:** K. Sakai, T. Moritaka, T. Morita, K. Tomita, T. Minami, T. Nishimoto, S. Egashira, M. Ota, Y. Sakawa, N. Ozaki, R. Kodama, T. Kojima, T. Takezaki, R. Yamazaki, S. J. Tanaka, K. Aihara, M. Koenig, B. Albertazzi, P. Mabey, N. Woolsey, S. Matsukiyo, H. Takabe, M. Hoshino, Y. Kuramitsu

**Affiliations:** 1 Graduate School of Engineering, Osaka University, 2-1 Yamadaoka, Suita, Osaka, 565-0871 Japan; 2 Institute of Laser Engineering, Osaka University, 2-6 Yamadaoka, Suita, Osaka, 565-0871 Japan; 3 Department of Helical Plasma Research, National Institute for Fusion Science, Toki, 509-5292 Japan; 4 Faculty of Engineering Sciences, Kyushu University, 6-1 Kasuga-Koen, Kasuga, Fukuoka, 816-8580 Japan; 5 Division of Quantum Science and Engineering, Graduate School of Engineering, Hokkaido University, Kita 13, Nishi 8, Kita-ku, Sapporo, Hokkaido, 060-8628 Japan; 6 Faculty of Engineering, University of Toyama, 3190 Gofuku, Toyama, Toyama, 930-8555 Japan; 7 Department of Physical Sciences, Aoyama Gakuin University, 5-10-1 Fuchinobe, Sagamihara, Kanagawa 252-5258 Japan; 8 LULI–CNRS, CEA, Sorbonne Universités, École Polytechnique, Institut Polytechnique de Paris, F-91120 Palaiseau cedex, France; 9 Department of Physics, York Plasma Institute, University of York, York, YO10 5DD UK; 10 Leung Center for Cosmology and Particle Astrophysics, National Taiwan University, Taipei, 10617 Taiwan; 11 Department of Earth and Planetary Science, University of Tokyo, 7-3-1 Hongo, Bunkyo, Tokyo 113-0033 Japan

**Keywords:** Laser-produced plasmas, Laboratory astrophysics, Magnetospheric physics, Solar physics

## Abstract

Magnetic reconnection is a universal process in space, astrophysical, and laboratory plasmas. It alters magnetic field topology and results in energy release to the plasma. Here we report the experimental results of a pure electron outflow in magnetic reconnection, which is not accompanied with ion flows. By controlling an applied magnetic field in a laser produced plasma, we have constructed an experiment that magnetizes the electrons but not the ions. This allows us to isolate the electron dynamics from the ions. Collective Thomson scattering measurements reveal the electron Alfvénic outflow without ion outflow. The resultant plasmoid and whistler waves are observed with the magnetic induction probe measurements. We observe the unique features of electron-scale magnetic reconnection simultaneously in laser produced plasmas, including global structures, local plasma parameters, magnetic field, and waves.

Magnetic reconnections are fundamental in various eruptive phenomena such as solar flares, coronal mass ejections, magnetic substorms, and disruptions of tokamak discharges in magnetically confined^1,2^, laser produced^3–9^, pulse power driven^10^, and space and astrophysical plasmas^11–16^, where the magnetic field energy is converted to the plasma energy and also changes the magnetic field topology^17^. The electron dynamics is considered to trigger the onset of magnetic reconnection; recent NASA’s Magnetospheric Multiscale (MMS) mission has revealed the electron-scale dynamics in magnetic reconnection^12–16^. The MMS spacecraft in the Earth’s magnetosphere provides insight into magnetic reconnection of the magnetic fields and the release of energy into the plasma and other electron-scale processes come from which are designed to resolve electron^12^. These processes include the formation of electron current sheets and outflows within an electron dissipation region^12–14^, and electron temperature anisotropy excitation of whistler waves^15,16,18^. The magnetic reconnection without coupling to ions is observed in the magnetosheath because of tiny spatial and temporal scales of turbulent plasmas^14^.The basic properties of “electron-only reconnection”, which can lead to Sweet-Parker reconnection in magnetohydrodynamics (MHD) limit, have been investigated^7,19–21^; numerical simulations show that the electron-only reconnection starts to transit to ion-coupled (MHD) reconnection at the spatial scale of $\gtrsim 10 d_{i}$, where $d_{i}$ is the ion skin depth^19^. The electron outflows close to the electron Alfvén velocity are observed^7,14,20,21^. These fast magnetic reconnection processes can be expressed as standing whistler waves (or standing kinetic Alfvén waves in the presence of a guide field), as MHD phenomena can be treated as a superposition of Alfvén waves^22–25^. Connecting these local multi-point observations to global information about the space plasma is challenging. On the other hand, global images are observed in astrophysical plasmas^11^ but electron-scale measurements are limited. We use laboratory experiments to observe both local and global information simultaneously in a controlled manner^26^.

In laser produced plasmas, magnetic reconnections have been studied using self-generated magnetic field by Biermann battery, which is an azimuthal magnetic field around the laser spot^3–6^. By irradiating a solid target with multiple laser beams, the azimuthal magnetic fields are advected with the plasma flow and anti-parallel magnetic fields collide and reconnect. The typical magnetic field strength and velocity are $\sim {1}\,\hbox {MG}$ and $\sim {100}\,\hbox {km/s}$, respectively^3^. The typical gyroradius is $\sim {10}\,\hbox {nm}$ for electron and $\sim {10}\,\upmu \hbox {m}$ for proton, therefore, the electron-scale is too tiny to resolve in the experiments and tends to be overlooked. Alternatively, there are experiments with external magnetic field using magnet^7^, pulsed power discharge^8^, and capacitor-coil target^9^. This allows us to control the parameters corresponding to the magnetic field such as gyroradius, gyrofrequency, and magnetization. We have used an external magnetic field strong enough to magnetize the electrons but not the ions. We briefly review our previous work^7^. In the previous work, the plasma collimation in the presence of a perpendicular external magnetic field is observed with interferometry, while there is no such collimation in the absence of the magnetic field^7^. The ion gyroradii estimated with the plasma flow velocity are much larger than the system size but electrons are well magnetized^7^. The plasma flow with dynamic pressure much larger than the magnetic pressure distorts the applied magnetic field, resulting in the charge separation across the magnetic field, since the electron is magnetized but ion is not. This leads to $\mathbf{E }\times \mathbf{B }$ drift only for electron. The electron moves along the distorted magnetic field rather than drift across the magnetic field and plasma is collimated. The collimation scenario is verified with particle-in-cell simulations^7,27^. The cusp and plasmoid propagation at electron Alfvén velocity with self-emission imaging indicates the magnetic reconnection at electron scale^7,28–30^. However, there was no observational evidence of the different motion between electron and ion, and the magnetic field relevant to reconnection event.

In this paper, we report the local observations of electron-scale magnetic reconnection in addition to global observations focusing on the electron dissipation region. The local velocity measurement clearly shows pure electron outflow that is not accompanied with the ion motion. The magnetic field measurements show the magnetic field inversion corresponding to the plasmoid, and also the whistler waves associated with electron-scale dynamics. The pure electron outflow demonstrates the magnetic energy is released to only the electrons on the onsets of the magnetic reconnection.

**Figure 1:**
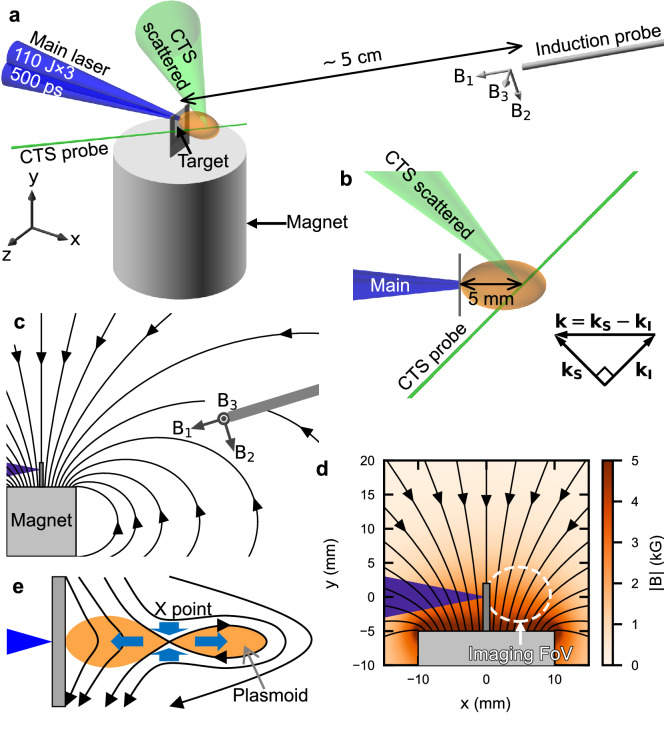
Experimental setup. (**a**) Schematic of the experiment. The three beams forming the main laser, shown in blue, irradiate a plastic (CH) foil target of thickness 10 $\upmu \hbox {m}$. The laser operates at a wavelength of 351 nm with pulse duration of 500 ps and 110 J/beam, the focal spot diameter is 300 $\upmu \hbox {m}$. Each focal spot is separated by 430 $\upmu \hbox {m}$ to obtain collimated plasma flow on the rear-side of the plastic target^31^. The laser propagates along the *x*-axis, the *y* and *z* axes define the orthogonal vertical and horizontal directions respectively. The target chamber is filled with either nitrogen gas of 5 Torr (case1) or e-4 Torr (case2). The upstream of target plasma is same in both cases. A permanent magnet placed below the target applies a static near-perpendicular magnetic field across the interaction region, with magnetic field components $(B_1,B_2,B_3)$ at the location of a three-axis induction probe placed at $\sim {5}\,\hbox {cm}$ from the target of $(43,19,0)~\hbox {G}$. This probe measures the time-dependent changes in the magnetic field ($\dot{B}_1,\dot{B}_2,\dot{B}_3$) is tilted at $({30}^\circ ,{73}^\circ ,{114}^\circ )$ with respect to the (*x*, *y*, *z*) axes as indicated by the gray arrows. (**b**) Top view of the experiment showing in green the CTS probe beam. This beam operates at a wavelength of 532 nm is focused 5 mm behind the target, with scattered light collected at ${90}^\circ$. The measured **k** is parallel to *x*, the main laser propagation direction. (**c**) The configuration of the initial magnetic field. (**d**) Enlarged view of (**c**). The magnetic field strength at the target is $\sim {3}\,\hbox {kG}$. The dashed white ellipse represents the field of view for imaging diagnostics. (**e**) Schematic illustration of the reconnection in our setup. The collimated plasma flow distorts the near-perpendicular magnetic field and forms the X point and plasmoid. The blue arrows indicate the inflows and outflows of the magnetic reconnection.

The experiment is performed with Gekko XII laser facility at Institute of Laser Engineering, Osaka University. The setup of the experiment and configuration of initial magnetic field are shown in Fig. 1, and the experimental details are found in the caption. We measure plasmas at the rear-side of the target. We use self-emission imaging as global diagnostics, and collective Thomson scattering (CTS)^32^ and a magnetic induction probe^33^ as local diagnostics. Figure 2a–c, d–f compare the measurements at 50 ns after the laser irradiation with and without an applied magnetic field, respectively. We obtain global information in Fig. 2a,d as well as local information in Fig. 2b,e simultaneously. The global images in Fig. 2a,d show collimated plasma flow originating from the main laser arriving from the left and interacting with a target at $(x,y)=(0,0)$ mm. The purple region in $0\lesssim x\lesssim 10$ mm and $-4\lesssim y\lesssim 4$ mm indicates emission from the resulting nitrogen plasma. The shocks result in bright emission regions centred near (9, 0) mm. Note that the increased emission at (5, 0) mm is due to the CTS probe beam interacting with the plasma and the plasmoid is smeared. Although the probe beam heats the plasma locally ionizing and increasing the electron density, the velocity is unchanged during this process. Figure 2b,e show CTS images in wavelength region of $532\lesssim \lambda \lesssim 532.4$ nm. The spectral profiles are plotted every 0.5 mm in Fig. 2c,f and fitted with scattering form factors^32,34–37^. The technical details are given in Supplementary information. Self-emission caused by CTS heating results in a near-constant background signal across all wavelengths at $-1.3\lesssim d\lesssim 0$ mm enabling this to be distinguished from other causes of emission. The wavelength shift from the probe wavelength is proportional to the ion flow velocity because of the Doppler shift by ion flows. The relation of wavelength shift and flow velocity is expressed as $\Delta \lambda /\lambda _0 = 2(v_i/c) \sin (\theta /2)$, where $\Delta \lambda$ is the shifted wavelength and $\theta$ is the scattering angle. Because the wavelength shift is $\sim {250}\,\hbox {pm}$ at $d={0}\,\hbox {mm}$ in Fig. 2c, the ion flow velocity is $\sim {100}\,\hbox {km/s}$. A red shift seen in the CTS spectrum indicates that the ions move in the positive *x* direction, i.e. along the laser propagation direction. In Fig. 2b,c, at position $d\sim 1.5$ mm (red spectrum), the spectrum shows an asymmetry about the shifted central wavelength. This asymmetry is not seen in the equivalent spectrum in Fig. 2e,f. This spectral feature suggests the electron velocity exceeds the ion velocity^32^, and that both move in the positive *x* direction. In contrast, the symmetric spectrum in Fig. 2e, the equivalent measurement with no applied magnetic field shows the electrons and ions move with the same velocity.

**Figure 2:**
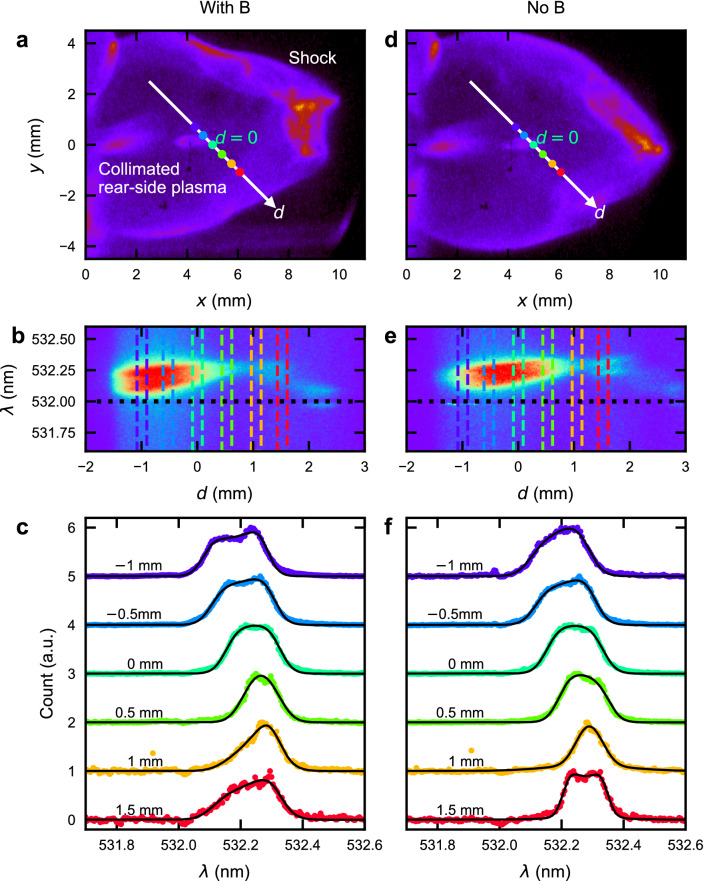
Local and global observations. Left (**(a–c)**) and right panels (**(d–f)**) are the measurements made with and without the applied magnetic field, respectively, in case1 taken 50 ns after the main laser interaction with the plastic foil target. (**a,d**) Transverse self-emission images. (**b,e**) CTS spectral images integrated over 2 ns, along direction *d* shown as a white arrow in (**a**) and (**d**). Note that *d* axis is actually in $x-z$ plane as shown in Fig. 1b. If the plasma is cylindrically symmetric about *x* axis, the arrow corresponds to the probe. The vertical dashed-coloured lines indicated a 175 $\upmu \hbox {m}$-wide regions used to extract the space-resolved spectral cross-sections shown in (**c**) and (**f**). The horizontal dashed-black line at 532 nm is at the wavelength of the probe. (**c,f**) The spectral cross-sections intensities are normalized to 1. The fitting results are shown in black curves.

**Figure 3:**
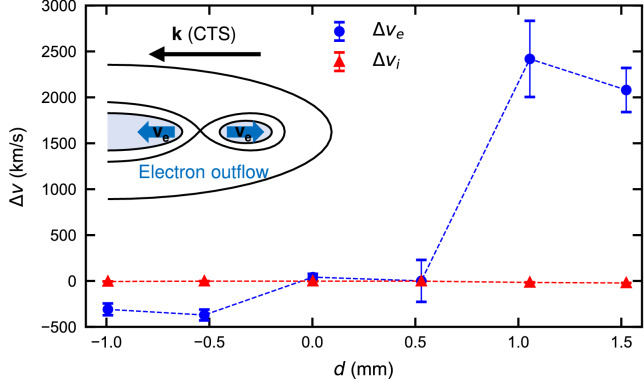
Velocity difference with and without applied magnetic field. We define $\Delta v_{e,i}\equiv v_{e,i}^\mathrm {B}-v_{e,i}^\mathrm {noB}$, where $v_{e,i}^\mathrm {B}$ is the velocity with the applied magnetic field and $v_{e,i}^\mathrm {noB}$ is without for electrons and ions, respectively. The blue and red markers are $\Delta v_{e,i}$ for electron and ion, respectively. The marked spatial change of $\Delta v_e$ is consistent with the electron outflow resulting from electron-scale magnetic reconnection as shown in the inset.

A plot of the velocity difference with and without the applied magnetic field is shown in Fig. 3. We define $\Delta v_{e,i}$ as the change in flow velocity by applying the external magnetic field. As the Biermann battery process self-generates a magnetic field and the reconnection by the Biermann battery magnetic field is also observed in our setup^6^, we compare the results in the presence and absence of the applied magnetic field in order to pick up the electron and ion motions related to the applied magnetic field (and the magnetic reconnection illustrated in Fig. 1e). Note that the reconnection outflows by the Biermann battery magnetic fields are perpendicular to the direction observed by CTS. This data shows that the differences in ion velocities are negligible, while there are significant spatial differences in the electron velocities. The analysis of the CTS spectra indicate ion velocities are not influenced by an applied magnetic field. The ion velocities are consistent with previous measurements made using a streaked optical pyrometer^7^. The electron velocity with the applied magnetic field is slower than that without the applied magnetic field at $d\lesssim 0$, whereas the electron changes the propagation direction of the relative velocity at $d\gtrsim 1$. This is an indication of pure electron outflow.

We estimate electron and ion gyroradii before the reconnection in ref.^7^, which shows the electrons are magnetized but not for ions. Here we estimate the relevant parameters after reconnection, electron and ion gyroradii [$$r_{g} = m v c/(q B)$$] and magnetization parameter [the ratio of a magnetic pressure to dynamic pressure $$\sigma = B^2/(4\pi n m v^2)$$]. Here *m*, *c*, *q*, and *n* are the mass, speed of light, charge, and the particle number density, respectively. The estimates use fits to the CTS spectra to infer ionization states of +1 for proton and +3 for carbon, typical flow velocities of 100 km/s, the electron temperature of 10 eV, and the ion temperature of 50 eV, the initial-at-target magnetic field of 3 kG, and the lowest electron density of $$10^{17}$$$${\hbox {cm}^{-3}}$$. We use the averaged velocity of $$v=(v_{flow}^2 + v_{th}^2)^{1/2}$$, where $$v_{flow}$$ and $$v_{th}$$ are the flow and thermal velocities, respectively. $$r_{ge} \sim {36}\,\upmu \hbox {m}$$ and $$\sigma _{e} \sim 0.22$$ for electron, $$r_{gp} \sim {4.9}\,\hbox {mm}$$ and $$\sigma _{p} \sim {8.7 \times 10^{-2}}{}$$ for proton, and $$r_{gc} \sim {14}\,\hbox {mm}$$ and $$\sigma _{c} \sim {1.3 \times 10^{-2}}{}$$ for carbon. Given the experiment is several millimeters in size (see Fig. 2a,d), it is clear that electrons are magnetized and the electron dynamics is coupled to the magnetic field dynamics. Note that, in principle, the earlier the timing from the laser irradiation is, the faster the plasma velocity in laser produced expanding plasmas (see Fig. 1 in ref.^7^). Before the reconnection, the ion gyroradii are much larger than the system size. Even after the reconnection, it is still larger than the observed reconnection region of $$\sim {2}\,\hbox {mm}$$. Since the ion skin depths for proton and carbon are $$d_p \sim 0.2$$ mm and $$d_c\sim 2$$ mm, respectively, the spatial scale of reconnection is on the order of the several skin depths and the magnetic reconnection can be electron-only^19^.

Because only the electrons are magnetized, the reconnected magnetic field pushes only the electron component of the plasma from the reconnection region. This occurs along the *x* axis as illustrated in the inset of Fig. 3. The difference in electron velocity is $\sim {2500}\,\hbox {km/s}$ in Fig. 3, which is twice of outflow velocity^7^. Using the measured electron density and initial magnetic field strength, the Alfvén velocity defined with the electron mass $m_e$ is $v_{Ae}=B/(4\pi n_e m_e)^{1/2}\sim 900$ km/s (note that we underestimate $v_{Ae}$ due to the overestimation of density). This leads to the conclusion that the spatial distributions in the velocity differences depicted in Fig. 3 result from pure electron outflow. The ions are not involved in magnetic reconnection process.

**Figure 4:**
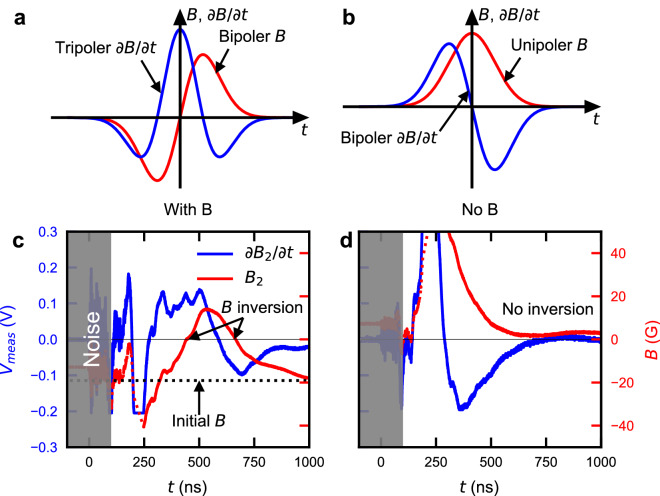
Local magnetic field inversion associated with plasmoid. (**a,b**) A schematic illustrating the relation between magnetic field (*B* in red) and time derivative of magnetic field ($\partial B/\partial t$ in blue) in bipolar and unipolar magnetic field, respectively. When the sign of magnetic field inverts (**a**), the signal of $\partial B/\partial t$ is tripolar. On the other hand, when a Biermann magnetic field approaches and passes through the probe (**b**), the signal of $\partial B/\partial t$ is bipolar. (**c,d**) Magnetic field measurements with and without the applied magnetic field in case2, respectively. The plots show the $B_2$ component where the magnetic field inversion is most significant. The blue and red curves represent the measured voltage and magnetic field, respectively. The velocity of fast plasma is $\sim 500$ km/s^7^, thus, the signal before 100 ns is attributed to be the electromagnetic noise. This region ($t<{100}\,\hbox {ns}$) is shaded gray. The voltage curves at $t\sim {200}\,\hbox {ns}$ briefly saturate. The magnetic field before the saturation is expressed as dotted red curves and our analysis likely underestimates the $B_2$ magnetic field before saturation. The dotted and solid horizontal lines represent the initial magnetic field strength and $B=0$, respectively. The voltage returns to 0 at the end of the trace. We integrate the signals from the end of time to avoid problems caused by noise and saturation at times before $\sim {250}\,{\hbox {ns}}$.

Figure 4 shows the local magnetic field measurements. The data were obtained in case2 in order for magnetic field to transport to the induction probe. In the presence of the shock wave with higher gas pressure (case1), the signal shows unique upstream wave feature (not shown). We focus on the magnetic reconnection where a plasmoid is generated and propagates toward the probe^7^. This is measured as a magnetic field inversion at the induction probe. In our experiment, Fig. 1, the magnetic field inversion is most significant in $B_2$ component. The plasmoid propagation velocity is $\sim {100}\,\hbox {km/s}$^7^ and the probe locates $\sim {5}\,\hbox {cm}$ away from the target, hence, the magnetic field inversion should occur around $t\sim ({5}\,\hbox {cm}) / ({100}\,\hbox {km/s}) = {500}\,{\hbox {ns}}$. The plasmoid velocity is close to the ion velocity measured with CTS. Although the electrons and ions move differently at the reconnection region, we assume that the electrons cannot be significantly apart from the ions at the probe where the spatial scale is several times larger than the ion gyroradius. The measured voltage (blue curves in Fig. 4c,d) is approximately proportional to the time derivative of the magnetic field. The magnetic field in Fig. 4d is likely the self-generated Biermann battery magnetic field^38^. While $B_1$ and $B_3$ are similar in both cases (see Supplementary Fig. 1), $B_2$ is considerably different with and without the applied magnetic field. The shape of blue curve in Fig. 4c,d are in qualitative agreement with that in Fig. 4a,b around $t\sim {500}\,{\hbox {ns}}$, respectively. This indicates the magnetic field inversion. We calculate the absolute value of the magnetic field in the red curves in Fig. 4c,d. It is clear that only $B_2$ in Fig. 4c is inverted at $t\sim 400$ and 700 ns. The magnetic field inversion can be understood as the propagation of the plasmoid or the low frequency magnetic fluctuation. If the inversion is a wave propagation, there should be the magnetic inversion not only in $B_2$ component but also in $B_3$ component (two components perpendicular to the background magnetic field). The magnetic field in $B_3$ component shows no inversion (see Supplementary Fig. 1c). Thus, the magnetic field inversion strongly indicates the passage of a plasmoid, the former and latter inversions correspond to the arrival and passage of plasmoid, respectively.

**Figure 5:**
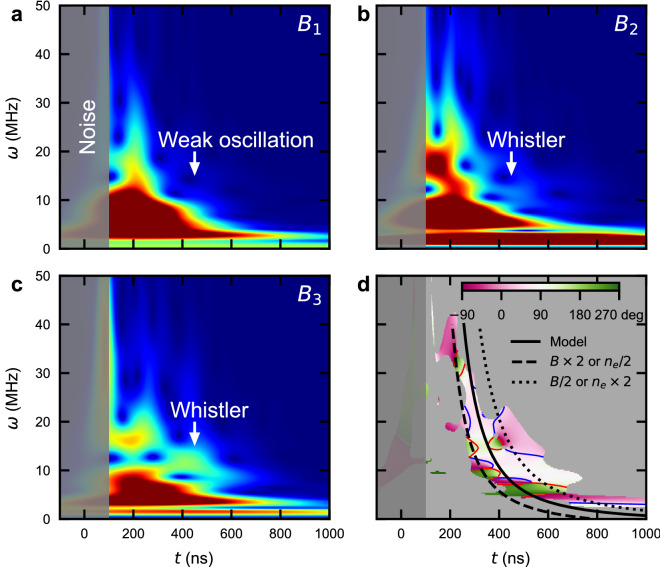
Whistler wave resulting from magnetic reconnection. (**a–c**) Time-frequency spectrogram of $B_1$-$B_3$ in Fig. 4c and Supplementary Fig. 1. As shown in Fig. 4, the electromagnetic noise is filled with gray. While there are distinct signals above 10 MHz at $t\sim 400$ ns in $B_2$ and $B_3$, the signal in $B_1$ is weak. According to the magnetic field strength in Supplementary Fig. 1, the magnetic field is almost parallel to $B_1$. (**d**) Phase difference of $B_2$ and $B_3$. We pick up the region where the signals are correlated with each other and they are not correlated with the dummy signal (see Supplementary Fig. 2). We fill the removed region with gray. The blue and red curves represent the contours of ${45}^\circ$ and ${135}^\circ$, respectively. The phase difference at the oscillation is $\sim {90}^\circ$. Because the frequency domain is between the electron and ion gyrofrequencies, the magnetic fluctuation is considered to be the whistler wave. We plot the whistler wave propagation model in black curves.

There is a oscillation of magnetic field at $t\sim 400$ ns, see Fig. 4c, when the applied magnetic field is present. We use wavelet analysis of the three magnetic field components, shown in Fig. 5a–c, to identify a $\sim 10$ MHz oscillation around $t\sim 400$ ns in the field components perpendicular to the nominal background magnetic field. The oscillation is only observed when the applied magnetic field is imposed as shown in Supplementary Fig. 2. This oscillation occurs in the range $\Omega _i < \omega \ll \Omega _e$, where $\Omega _{i}$ is the ion gyrofrequency at $\sim 1$ MHz. The phase difference of $B_2$ and $B_3$ in Fig. 5d shows $\sim {90}^\circ$, which corresponds to right-hand polarization. The oscillation is recognized as the whistler wave.

The higher and lower frequencies of the whistler wave in Fig. 5 propagate faster and slower, respectively. We model the timing at which the whistler wave arrives at the probe in the presence of expanding plasma. The wave propagation velocity in the laboratory frame is sum of plasma velocity (*u*) and group velocity of the whistler wave ($v_g$). We simplify to one-dimension propagation. We assume the expansion velocity of plasma as $u=s/t$, where *s* is the position of wavefront from the target, and *t* is the time after the laser irradiation. Therefore, the wave propagation velocity is expressed as $ds/dt = (s/t) + v_g$. Solving the differential equation, we obtain the arrival timing of whistler wave. The initial conditions are $s=0$ mm and $t=50$ ns from CTS results. In the frequency range of $\Omega _i \ll \omega \ll \Omega _e, \omega _{pe}$, the group velocity is approximated to $v_{g}/c = 2 (\omega \Omega _{e})^{1/2}/\omega _{pe} \propto (\omega B/n_{e})^{1/2}$, where $\omega _{pe}$ is the plasma frequency. This shows that the group velocity is determined by $n_e/B$. As only the electrons are magnetized we assume that $n_e/B$ is near constant in the plasmoid, although the electron dynamics can change the density and magnetic field change in time. The value for $n_e/B$ at the reconnection region uses $n_{e0} \sim 1\times 10^{17}~\mathrm {cm^{-3}}$ measured with CTS, and the initial strength of $B_0 \sim 3$ kG. The density is likely overestimated as the CTS probe beam ionizes the plasma. The black curves in Fig. 5d show the predictions from the model for the arrival time of whistler waves with the range of $B_{0}/(2n_{e0}) \le B/n_{e} \le 2 B_{0}/n_{e0}$. These qualitatively match the $\sim {90}^\circ$ phase difference region and illustrate that these oscillations are whistler waves.

In summary, we report the local observations of magnetic reconnections driven by electron dynamics in laser produced plasmas. The local velocity measurements directly reveal the pure electron outflow occurs at both sides of a reconnection region. Magnetic reconnection generates a plasmoid or magnetic island. The local magnetic field measurements show the magnetic field inverts twice, this corresponds to the passage of a plasmoid, and the whistler waves resulting from electron-scale dynamics. The electron outflow, magnetic field inversion, and resultant whistler waves are the direct evidences of electron-scale magnetic reconnection. We showed the electron dynamics governing macroscopic phenomena of magnetic reconnection in laser produced plasmas. This indicates the magnetic energy is converted to only the electrons on the onsets of the magnetic reconnection.

Our experimental results provide simultaneous measurements of global structures, local plasma parameters, magnetic fields, and waves in a controlled manner. In the presence of whistler waves, the electrons can be further accelerated by the cyclotron resonance. The next milestone is the direct observation of nonthermal electron acceleration by the whistler waves. We are developing diagnostics to intrinsically measure the wave growth^39,40^, leading to identify the excitation location and timing of whistler and other waves. The recent 3D simulation shows the reconnection rate increases as a result of localized reconnection region^41^. The 3D reconnection rate can be observed using multi-channel CTS measurements or electric/magnetic field measurements at the reconnection region with proton radiography^4,5,8^. Moreover, the experiment can be extended to relativistic regime using ultraintense laser pulses^42,43^ and turbulent regime using multiple beams^44,45^. Laboratory experiments will contribute further understanding the magnetic reconnections.

## Supplementary Information

Supplementary Information.

## Data Availability

The data that support the findings of this study are available from the corresponding author upon reasonable request.
