# Author Correction: Direct observations of pure electron outflow in magnetic reconnection

**DOI:** 10.1038/s41598-022-21220-5

**Published:** 2022-10-03

**Authors:** K. Sakai, T. Moritaka, T. Morita, K. Tomita, T. Minami, T. Nishimoto, S. Egashira, M. Ota, Y. Sakawa, N. Ozaki, R. Kodama, T. Kojima, T. Takezaki, R. Yamazaki, S. J. Tanaka, K. Aihara, M. Koenig, B. Albertazzi, P. Mabey, N. Woolsey, S. Matsukiyo, H. Takabe, M. Hoshino, Y. Kuramitsu

**Affiliations:** 1 Graduate School of Engineering, Osaka University, 2‑1 Yamadaoka, Suita, Osaka 565‑0871 Japan; 2 Institute of Laser Engineering, Osaka University, 2‑6 Yamadaoka, Suita, Osaka 565‑0871 Japan; 3 Department of Helical Plasma Research, National Institute for Fusion Science, Toki, 509‑5292 Japan; 4 Faculty of Engineering Sciences, Kyushu University, 6‑1 Kasuga‑Koen, Kasuga, Fukuoka 816‑8580 Japan; 5 Division of Quantum Science and Engineering, Graduate School of Engineering, Hokkaido University, Kita 13, Nishi 8, Kita‑ku, Sapporo, Hokkaido 060‑8628 Japan; 6 Faculty of Engineering, University of Toyama, 3190 Gofuku, Toyama, Toyama 930‑8555 Japan; 7 Department of Physical Sciences, Aoyama Gakuin University, 5‑10‑1 Fuchinobe, Sagamihara, Kanagawa 252‑5258 Japan; 8 LULI–CNRS, CEA, Sorbonne Universités, École Polytechnique, Institut Polytechnique de Paris, 91120 Palaiseau Cedex, France; 9 Department of Physics, York Plasma Institute, University of York, York, YO10 5DD UK; 10 Leung Center for Cosmology and Particle Astrophysics, National Taiwan University, Taipei, 10617 Taiwan; 11 Department of Earth and Planetary Science, University of Tokyo, 7‑3‑1 Hongo, Bunkyo, Tokyo, 113‑0033 Japan

Correction to: *Scientific Reports* 10.1038/s41598-022-14582-3, published online 30 June 2022

In the original version of this Article, Y. Kuramitsu was omitted as a corresponding author. Correspondence and requests for materials should also be addressed to kuramitsu@eei.eng.osaka-u.ac.jp.

In addition, the original version of this Article contained errors in the main text.

“The estimates use fits to the CTS spectra to infer ionization states of +1 for proton and +3 for carbon, typical flow velocities of 100 km/s, the electron temperature of 10 eV, and the ion temperature of 50 eV, the initial-at-target magnetic field of 3 kG, and the lowest electron density of $\text{e}17\,{\mathrm{cm}}^{-3}$.”

now reads:

“The estimates use fits to the CTS spectra to infer ionization states of +1 for proton and +3 for carbon, typical flow velocities of 100 km/s, the electron temperature of 10 eV, and the ion temperature of 50 eV, the initial-at-target magnetic field of 3 kG, and the lowest electron density of $10^{17}\,{\mathrm{cm}}^{-3}$.”

And,

$r_{ge} \sim 36\,\upmu {\text{m}}$ and $\sigma_{e} \sim 0.22$ for electron, $r_{gp} \sim 4.9\,{\text{mm}}$ and $\sigma_ {p} \sim 8.7e - 2$ for proton, and $r_{gc} \,14\,\,{\text{mm}}$ and $\sigma_{ c} \sim 1.3e - 2$ for carbon.

now reads:

$r_{ge} \sim 36\,\upmu {\text{m}}$ and $\sigma_ {e} \sim 0.22$ for electron, $r_{gp} \sim 4.9\,{\text{mm}}$ and $\sigma_{ p} \sim 8.7 \times 10^{ - 2}$ for proton, and $r_{gc} \,14\,\,{\text{mm}}$ and $\sigma_ {c} \sim 1.3 \times 10^{ - 2}$ for carbon.

The original Article has been corrected.

