# Supplementary material for: Direct observations of pure electron outflow in magnetic reconnection
Source: Sci Rep. 2022 Jun 30;12:10921. doi: 10.1038/s41598-022-14582-3 (PMC9247195; doi:10.1038/s41598-022-14582-3)
Supplement: Supplementary file 1 — Supplementary Information. [file 41598_2022_14582_MOESM1_ESM.pdf]

Supplementary information

Direct observations of pure electron outflow in magnetic reconnection

K. Sakai *et al.*

Collective Thomson scattering analysis

We fit the CTS spectra in Figs. 2c,f with the scattering form factor to estimate the local velocities of electron and ion [1]. The spatial resolution of the CTS spectrometer is considered in the spectral fitting [2]. We averaged the spectra in the spatial direction. The configuration of the spectrometer is same as ref. [3]. The diffraction limit of the CTS collection system is $\sim 4\,\mu m$ that is less than the camera's resolution of $\sim 20\,\mu m$ (Princeton Instruments, PI-MAX4). Because these resolutions are smaller than the width of $175\,\mu m$ to extract the averaged CTS spectra, the finite aperture effect is not serious.

The ion feature of CTS is a parametric resonance among incident electromagnetic, scattered electromagnetic, and ion acoustic waves. The two peaks of ion feature show the amplitudes of ion acoustic wave that satisfies the phase matching condition. Since the dispersion relation for ion acoustic wave is $\omega = \pm c_s k$, where c_s is the sound velocity in a plasma, the frequency of scattered wave shifts by $\pm c_s k$ and this leads to the red-shifted and blue-shifted peaks. As a result of Landau damping, the amplitude of ion acoustic wave decreases with the damping rate proportional to the derivative of electron distribution function at the phase velocity of the ion acoustic wave. When the derivatives of electron distribution function corresponding to two peaks are equal, i.e., the electron and ion flow velocities are same, the CTS spectrum shows symmetric feature. On the other hand, when the electron velocity exceeds the ion velocity, the damping rate in the blue-shifted peak is higher than that in the red-shifted peak, resulting in the spectral asymmetry. By combining the velocity difference with the ion flow velocity measured using the Doppler shift, the electron flow velocity is obtained.

The superpositions across the temperature, density, and velocity gradients along the probe beam can also affect the spectra and mainly overestimate the temperature as a result

of broaden spectra. For instance, the central wavelength spatially changes in Figs. 2b,e due to the velocity gradient. Since the central wavelength shifts by $\sim 100\,\mathrm{pm}$ at $-1 \leq d \leq 1\,\mathrm{mm}$ in Fig. 2b, the change in central wavelength is $\sim 10\,\mathrm{pm}$ for the $175\,\mu\mathrm{m}$-width and broaden the averaged spectra. The ion temperature can be overestimated by $\sim 0.1\,\mathrm{eV}$. This is much smaller than the estimated error of ion temperature $\lesssim 10\,\mathrm{eV}$. When the gradients are large, e.g., the shock transition region at $d \sim 1.8\,\mathrm{mm}$ in Fig. 2b, it is almost impossible to estimate parameters from the observed spectra. In the present analysis, we choose the position where the profile does not change drastically in the width to average the spectra.

We fit the results using the scattering form factor convolved with the spectra of Rayleigh scattering, which gives the instrument function [2]. The wavelength resolution of CTS spectrometer is $\sim 10\,\mathrm{pm}$. This is enough to resolve the spectra in Fig. 2c. Since the ion temperature is relatively higher than electron one, the peaks of ion acoustic wave are broad, however, it does not mean poor resolution but the spectrometer resolves the higher ion temperature than electrons. This is commonly observed in laser produced plasmas where the electron loses the energy by self-emission [3, 4]. In the presence of shock wave, e.g. $d \sim 2\,\mathrm{mm}$ in Fig 2b, the ion temperature becomes high at the downstream and we can observe the clear double peak spectrum [2].

Another potential source of asymmetry in spectra is non-Maxwellian distribution function of Spitzer-Härm in the presence of heat flux [5, 6]. The higher energy electron carries the heat counter to the temperature gradient and the lower energy electron moves opposite to the higher one to maintain the charge neutrality in the Spitzer-Härm electron distribution. Henchen et al. (2018) [5] observed the asymmetry $\sim 1\,\mathrm{mm}$ away from the target at $t \sim 1\,\mathrm{ns}$ in the electron feature of CTS. In our setup, the measured location is $\sim 5\,\mathrm{mm}$ and the timing is $50\,\mathrm{ns}$. As shown in Fig. 3 in ref. [5], the heat flux decreases spatially. The heat flux can be negligible at $5\,\mathrm{mm}$ away from the target by extrapolating the figure. When the heat flux is measured with CTS, the temperature gradient is $\sim 100\,\mathrm{eV}/100\,\mu\mathrm{m}$ in Fig. 4 in ref. [5], that is three orders of magnitude larger than the temperature gradient of $\sim 10\,\mathrm{eV}/1\,\mathrm{mm}$ in our experiment. Therefore, it is unlikely that the observed asymmetry originates from the heat flux.

While distribution functions that are currently considered in CTS analysis, such as Spitzer-Härm distribution, are time-independent, temporal evolution in distribution function may change the shape of observed spectrum. Although we have investigating CTS in

non-equilibrium plasmas where the distribution function varies in time [7, 8], the analysis is still unestablished. We consider here a standard analysis where the stable distribution function with the relative drift between electron and ion as a best analysis that can be done now [1].

Magnetic induction probe

We obtain voltage relevant to the derivative of magnetic field with the magnetic induction probe. The relation between the magnetic field and measured voltage V_{meas} is [9]

$$aNg\frac{dB}{dt} = \left[1 + \tau_s \frac{d}{dt}\right] V_{meas}, \tag{1}$$

where a, N, g, and τ_s are the cross-sectional area of the coil, number of the loops, gain of the differential amplifier, and relaxation parameter, respectively. The parameters of the probe is shown in Supplementary Table **1**. Integrating Eq. (1) from the end of the signal ($t = T$), we obtain

$$B(t) = B(T) + \frac{\tau_s}{aNg}\left[V_{meas}(t) - V_{meas}(T)\right] - \frac{1}{aNg}\int_t^T V_{meas} dt. \tag{2}$$

The magnetic field is calculated with Eq. (2).

We plot all the data of magnetic field measurement in Supplementary Fig. **1**. Comparing the blue curves with and without the applied magnetic field, B_1 and B_3 components are similar, while B_2 component are different. The shapes of blue curves are not tripolar (see Fig. 4a) around $t = 500$ ns except Supplementary Fig. **1**b. The red curve crosses $B = 0$ at $t \sim 500$ ns only in Supplementary Fig. **1**b. The magnetic field inversion in B_2 with the applied magnetic field is relevant to the plasmoid. The magnetic field in Supplementary Figs. **1**e-g are considered to be the magnetic field via the Biermann battery effect [10–13]. Because the Biermann magnetic field is generated in the direction of $\nabla T_e \times \nabla n_e$, the

Supplementary Table 1. Parameters of the magnetic induction probe.

	B_1	B_2	B_3
a (mm^2)	2.16 ± 0.01	2.76 ± 0.01	3.27 ± 0.01
N		4	
g		0.5	
τ_s (ns)	49 ± 1	50 ± 1	44 ± 1

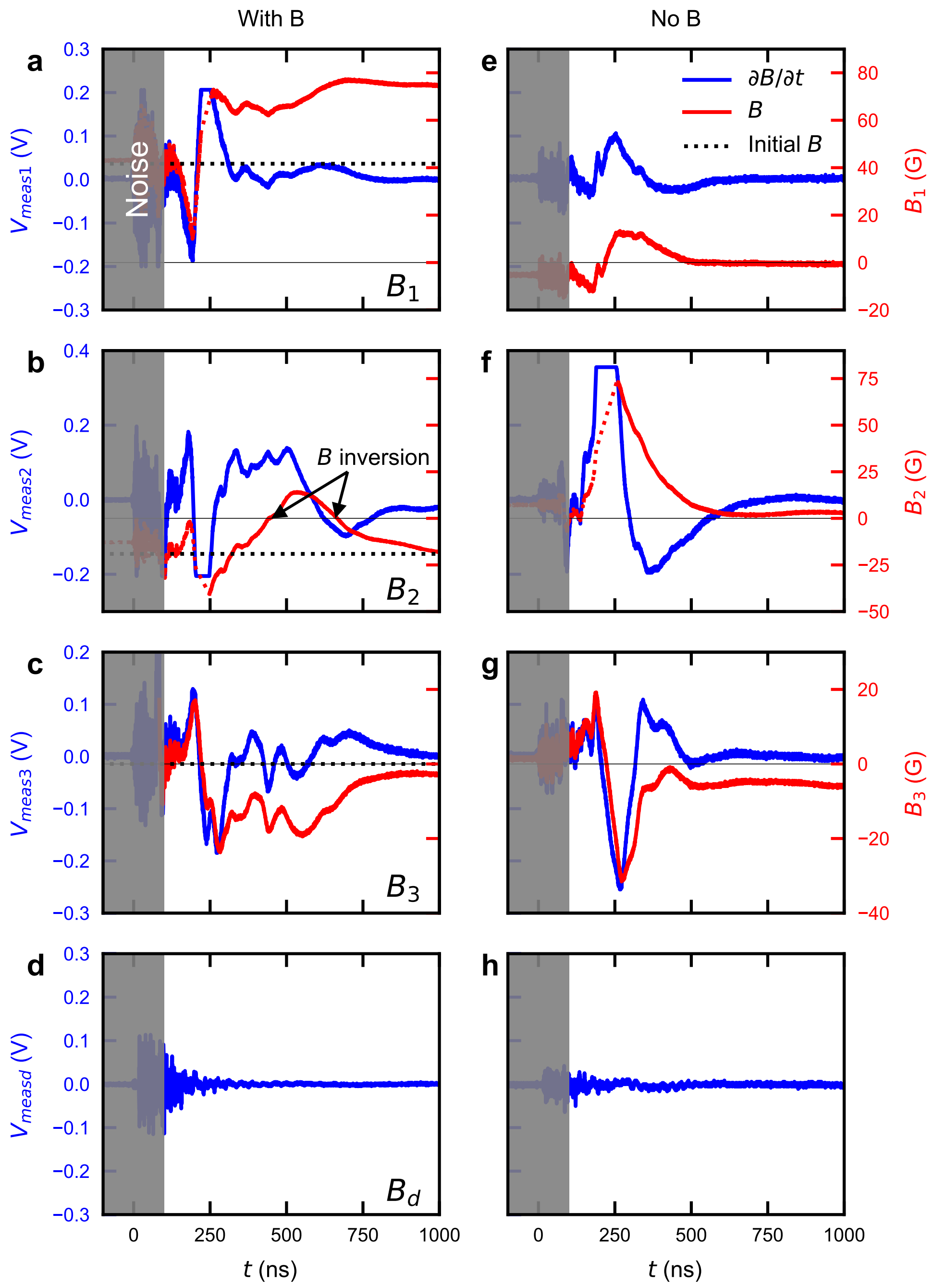

Supplementary Figure 1. **All the components of induction probe.** Left panels **(a-d)** and right panels **(e-h)** are the results with and without the applied magnetic field, respectively. **a,e**, **b,f**, **c,g**, and **d,h** represent the B_1, B_2, B_3, and dummy (B_d) components, respectively. Blue curves denote the measured voltages, and red curves denote the magnetic field strength. The dotted and solid horizontal lines represent the initial magnetic field strength and $B = 0$, respectively.

magnetic field is relatively weak in B_1 component, positive in B_2 component, and negative in B_3 component. The red curves in Supplementary Figs. 1e-g show this characteristics. The magnetic field passes through until ~ 500 ns. The dummy components in Supplementary Figs. 1d,h have no loops, thus, the signal is recognized as a noise. Because the noises are much weaker than the signals in Supplementary Figs. 1a-c,e-g, the signals correspond to the magnetic field.

Wavelet analysis of magnetic field

We perform the wavelet transform in order to obtain time-frequency spectrogram. We use the Morlet wavelet as a mother wavelet. Supplementary Figs. 2a-d and e-h show the magnetic field spectrogram with and without the external magnetic field, respectively. All the signals in Supplementary Figs. 2a-c,e-g are significantly stronger than the dummy data in Supplementary Figs. 2d,h, and thus, the signals show the magnetic field. Comparing the results with and without the external magnetic field, there are little signals without the external magnetic field in the region corresponding to the whistler waves in Supplementary Figs. 2b,c. While the amplitude and sign of the Biermann magnetic field are different among the components in Supplementary Figs. 1e-g, the magnetic field contains similar frequencies in all the components as shown in Supplementary Figs. 2e-g.

[1] Froula, D. H., Glenzer, S. H., Luhmann, N. C. & Sheffield, J. *Plasma Scattering of Electromagnetic Radiation: Theory and Measurement Techniques* (Academic Press, Amsterdam, 2011), 2nd edn.

[2] Morita, T. *et al.* Thomson scattering measurement of a shock in laser-produced counter-streaming plasmas. *Physics of Plasmas* **20**, 092115 (2013).

[3] Bolouki, N. *et al.* Collective thomson scattering measurements of electron feature using stimulated brillouin scattering in laser-produced plasmas. *High Energy Density Physics* **32**, 82–88 (2019).

[4] Kuramitsu, Y. *et al.* Time evolution of Kelvin–Helmholtz vortices associated with collisionless shocks in laser-produced plasmas. *The Astrophysical Journal* **828**, 93 (2016).

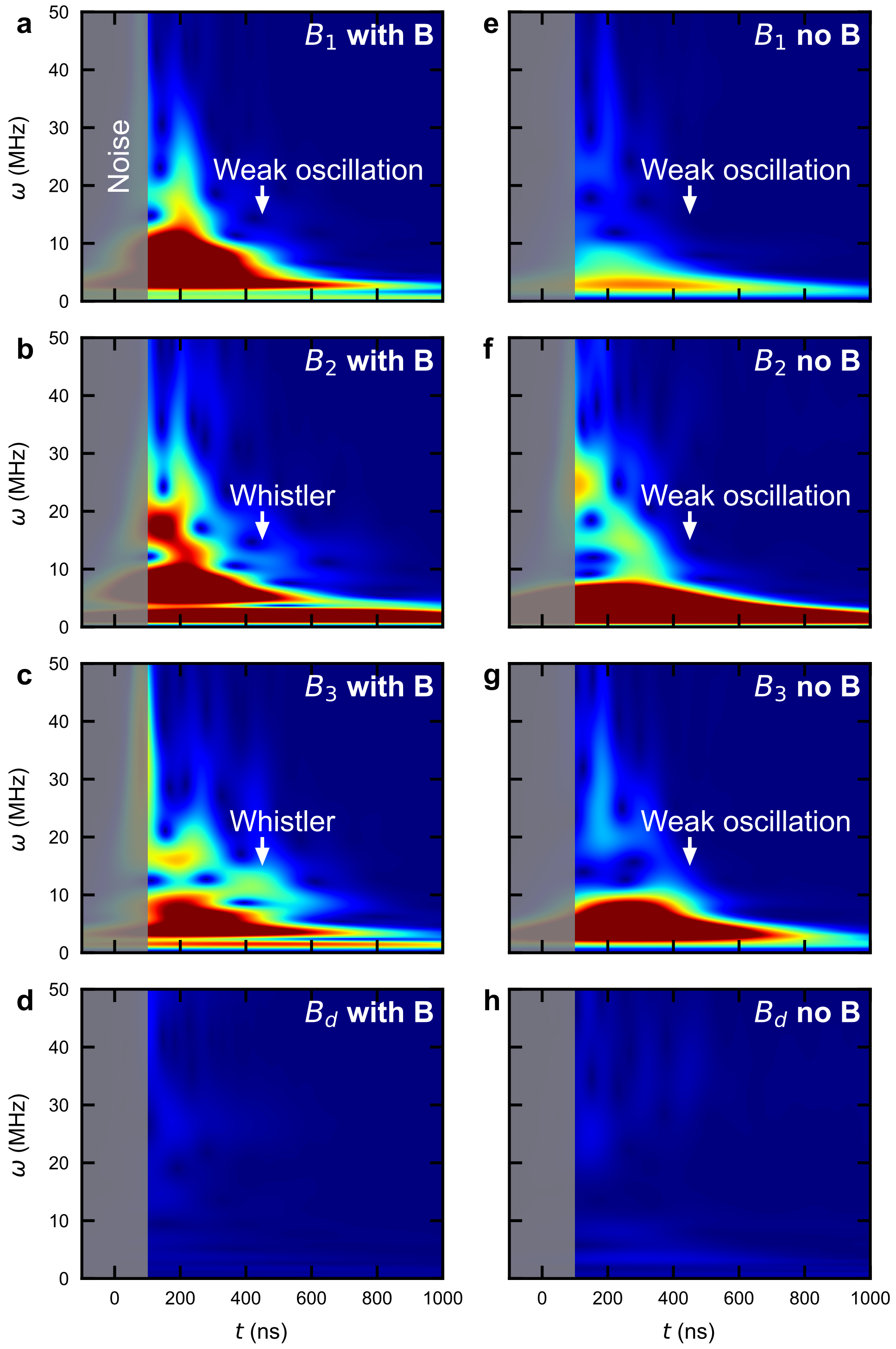

Supplementary Figure 2. **Wavelet analysis.** Left panels **(a-d)** and right panels **(e-h)** are the time-frequency spectrograms with and without the external magnetic field, respectively. **a,e**, **b,f**, **c,g**, and **d,h** represent the B_1, B_2, B_3, and B_d components, respectively. **a-c** are the same plot as Figs. 5a-c.

[5] Henchen, R. J. *et al.* Observation of nonlocal heat flux using thomson scattering. *Phys. Rev. Lett.* **121**, 125001 (2018).

[6] Henchen, R. J. *et al.* Measuring heat flux from collective thomson scattering with non-maxwellian distribution functions. *Physics of Plasmas* **26**, 032104 (2019).

[7] Matsukiyo, S., Kuramitsu, Y. & Tomita, K. Collective scattering of an incident monochromatic circularly polarized wave in an unmagnetized non-equilibrium plasma. *Journal of Physics: Conference Series* **688**, 012062 (2016).

[8] Sakai, K. *et al.* Collective thomson scattering in non-equilibrium laser produced two-stream plasmas. *Physics of Plasmas* **27**, 103104 (2020).

[9] Everson, E. T. *et al.* Design, construction, and calibration of a three-axis, high-frequency magnetic probe (B-dot probe) as a diagnostic for exploding plasmas. *Review of Scientific Instruments* **80**, 113505 (2009).

[10] Zhong, J. *et al.* Modelling loop-top X-ray source and reconnection outflows in solar flares with intense lasers. *Nature Physics* **6**, 984–987 (2010).

[11] Nilson, P. M. *et al.* Magnetic reconnection and plasma dynamics in two-beam laser-solid interactions. *Phys. Rev. Lett.* **97**, 255001 (2006).

[12] Li, C. K. *et al.* Observation of megagauss-field topology changes due to magnetic reconnection in laser-produced plasmas. *Phys. Rev. Lett.* **99**, 055001 (2007).

[13] Gregori, G. *et al.* Generation of scaled protogalactic seed magnetic fields in laser-produced shock waves. *Nature* **481**, 480–483 (2012).
